# Use of extended criteria donor cardiac allografts after multiple offers is associated with inferior post-transplant outcomes^1^

**DOI:** 10.1016/j.jhlto.2026.100566

**Published:** 2026-04-16

**Authors:** Cindy Song, Amit Iyengar, David Rekhtman, Michaela Asher, Max Shin, Noah Weingarten, Omar Toubat, Mauer Biscotti, Marisa Cevasco

**Affiliations:** a Division of Cardiovascular Surgery, Hospital of the University of Pennsylvania, Philadelphia, PA; b Perelman School of Medicine, University of Pennsylvania, Philadelphia, PA

**Keywords:** Extended criteria donor, Donor sequence number, Heart transplant, Donor selection

## Abstract

**Purpose:**

Extended criteria donor (ECD) cardiac allografts have increased the donor pool for heart transplant (HT). The effect of donor sequence number (DSN), number of times a recipient’s donor organ has been offered prior to acceptance, on post-ECD HT outcomes is unknown.

**Methods:**

Adults undergoing first-time isolated HT using ECD from 2012 to 2022 were identified in the United Network for Organ Sharing database. ECD was defined as donor age ≥55 years, ejection fraction (EF) <50%, left ventricular or septal wall thickness >11 mm, valve or wall motion abnormality, abnormal catheterization, or size mismatch >20%. Patients were stratified by DSN, forming 3 groups: 1–5, 6–10, and >10.

**Results:**

12,438 patients underwent ECD HT. 8374 (67%) donors were accepted after 1–5 offers, 1537 (12%) after 6–10 offers, and 2527 (20%) after >10 offers. Compared to those with DSN >10, recipients with DSN 1–5 were more likely to be of high listing status (status 1A or 1–2) (72% vs. 37%, p<0.001) and require waitlist life support (88% vs. 76%, p<0.001). Compared to DSN ≤10, DSN >10 was associated with lower 3-year graft survival estimates (82% vs. 85%, log-rank p=0.011), largely driven by the subgroup of donors with EF <50%. Single-unit increases in DSN were not associated with significantly increased predicted hazard of 3-year graft failure (average marginal effect 0.0003 [−0.0007 – 0.0012], p=0.550).

**Conclusions:**

Although there is an association between high DSN and inferior post-transplant outcomes in ECD HT, large differences in DSN are needed to observe meaningful increase in graft failure.

## Graphical abstract

**Figure:**
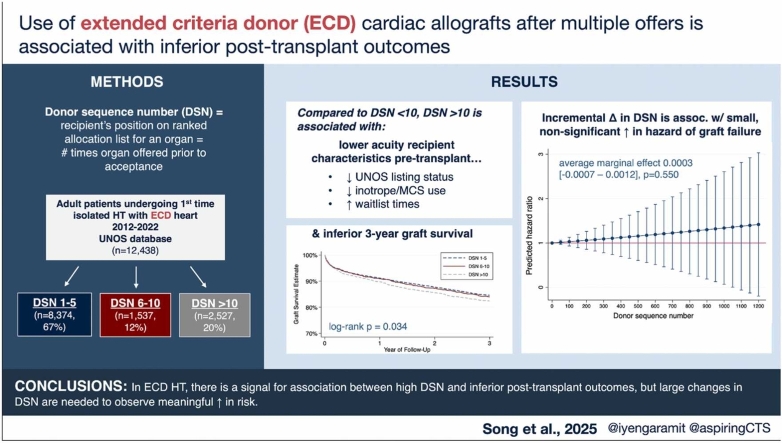

## Introduction

Heart transplant (HT) is the gold standard treatment for patients with end-stage heart failure, offering the best long-term survival and quality of life. However, demand for suitable donor hearts far outweighs the available supply.^1^ Donor pool expansion is an important strategy to address the donor-recipient imbalance, particularly given the high rates of donor heart nonuse. In the United States (US), an estimated 60–65% of potential donors are ultimately not used, most commonly due to older age, female sex, medical comorbidities, and markers of cardiac dysfunction.^1^, ^2^ Use of extended criteria donor (ECD) cardiac allografts, which have one or more characteristics traditionally perceived to confer higher risk of post-transplant morbidity and mortality, is a growing strategy to increase donor utilization. In the right clinical setting and candidate, outcomes after ECD HT appear acceptable compared to standard HT, and are likely better than the natural history of end-stage heart failure for patients who may have otherwise died on the waitlist or not been a transplant candidate.^3^, ^4^, ^5^ However, management of ECD HT remains more complex than standard HT, so careful donor-recipient matching is critical to optimize outcomes.^6^, ^7^

When a donor organ becomes available, the United Network for Organ Sharing (UNOS) generates a “match run”, a ranked list of recipients that the allograft will be offered to based on medical urgency, distance, and blood type/size matching.^8^ After receiving an organ offer, the transplant team has the opportunity to review donor information and then accept or reject the offer. Since 2007, transplant centers are able to view the candidate’s donor sequence number (DSN), which represents the number of times the donor organ has been declined for other potential recipients that were positioned higher on that organ’s ranked allocation list.^9^ Prior studies evaluating all HT recipients have concluded that higher DSN does not independently increase risk of post-transplant mortality.^9^, ^10^ However, the effect of DSN on post-transplant outcomes in recipients of ECD allografts, which by definition are more likely to be of suboptimal quality, remains unknown. We sought to investigate whether use of ECD allografts that were previously declined many times prior to acceptance (i.e. high DSN) predicts inferior post-transplant outcomes.

## Materials and methods

### Ethical statement

Due to use of deidentified data, the need for written informed consent was waived by the University of Pennsylvania institutional review board. This study was performed in compliance with the International Society for Heart and Lung Transplantation (ISHLT) ethics statement.

### Study design and population

This was a retrospective cohort study of adult patients undergoing first-time isolated HT with an ECD heart between January 1, 2012 and December 31, 2022 using the UNOS Thoracic database. The UNOS database was queried for patient demographics, pre-operative characteristics, donor and transplant characteristics, and post-operative outcomes. As previously described, definition of ECD was based on failure to meet the criteria for acceptable donor hearts described in the 2020 ISHLT Consensus Statement on Donor Heart and Lung Procurement.^11^, ^12^ Thus, ECD was defined as donor age ≥55 years, left ventricular ejection fraction (LVEF) <50%, left ventricular (LV) or septal wall thickness >11 mm, valve abnormality, wall motion abnormality, abnormal cardiac catheterization, or weight-based size mismatch >20%. Match run data from the UNOS Potential Transplant Recipient file was linked to obtain DSNs. Patients were stratified by number of offers prior to donor organ acceptance (DSN), forming 3 distinct groups: 1–5, 6–10, and >10 offers. To identify inflection points in effect of DSN on graft failure, DSN was modeled in a Cox proportional-hazards model using restricted cubic splines with 3 knots placed at the 10th, 50th and 90th percentiles of the log-transformed distribution; extreme DSN values were truncated to reduce instability. The primary outcome was 3-year graft failure, defined as death or re-transplantation.

### Statistical analysis

Descriptive statistics were presented as medians with interquartile ranges for continuous data or counts with frequencies for categorical data. Groups were compared using Kruskal-Wallis tests for continuous variables and chi-square tests for categorical variables. Pairwise deletion was used to account for missingness. Missingness of all variables, excluding donor echocardiographic and catheterization data and 1-year post-transplant rejection, was <15% (Table S1).

Time-to-event analyses were performed using Kaplan-Meier estimation, censored at 3 years post-transplant. Log-rank tests were used to determine statistical significance between groups. Cox proportional-hazards models for 3-year graft failure were developed. Candidate variables included pre-operative recipient, donor, and transplant characteristics selected based on clinical expertise. Variables with univariable p<0.4 for 3-year graft failure were included in development of the final multivariable model, which was created using backward elimination with an exclusion criterion of p<0.2. Univariable Cox regressions for all predictor variables can be found in Table S2.

To visualize effect of DSN on hazard of 3-year graft failure, predictive margins and average marginal effects were calculated from univariable and multivariable Cox models of 3-year graft failure. Covariates for adjustment were obtained from the final Cox multivariable model, developed as described above. Baseline hazard for predictive margins was set at 1 by centering continuous variables at their means and holding discrete variables at their reference values. All analyses were performed using Stata/BE 17.0 (StataCorp, College Station, TX).

## Results

### Study population

**Figure 1:**
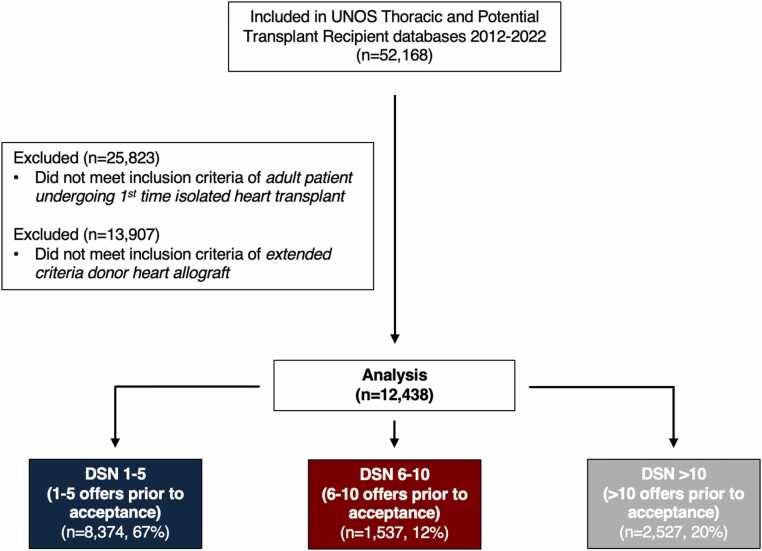
Patient inclusion and exclusion criteria. DSN, donor sequence number; UNOS, United Organ for Network Sharing.

**Figure 2:**
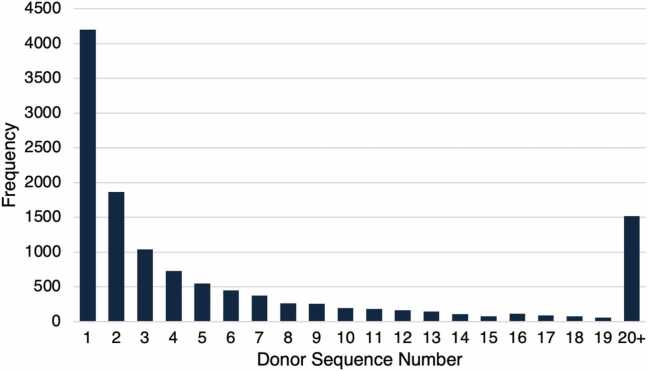
Number of extended criteria heart transplants by donor sequence number.

During the 10-year study period from 2012 to 2022, 12,438 patients underwent first-time isolated HT using an ECD allograft. Of these, 8374 (67%) donor hearts were accepted after 1–5 offers, 1537 (12%) after 6–10 offers, and 2527 (20%) after >10 offers (Figure 1). DSN ranged from 1 to 1218, with a median of 3 (interquartile range 1–8) (Figure 2). Restricted cubic spline modeling demonstrated an inflection region at DSN 4–7 (Figure S1).

#### Donor characteristics

In the overall cohort, size mismatch was the most commonly met ECD criterion (positive in 76.3% of donors), followed by thickened septal (17.8%) and LV walls (17.7%), coronary artery disease (CAD) (7.40%), age ≥55 years (5.5%), LVEF <50% (3.1%), wall motion abnormality (1.5%), and valve abnormality (0.4%) (Figure S2). Most ECD criteria were more commonly met in donor organs that were accepted after >10 offers, compared to 1–5 offers (Table 1). However, the opposite trend was true for size mismatch (Table 1). The proportion of donors meeting multiple ECD criteria increased with DSN group (Table 1). Donors that died of trauma were more likely to be accepted after 1–5 offers than >10 offers, while those that died of stroke or cardiovascular etiologies were more likely to be offered >10 times prior to acceptance (Table 1).

**Table 1:** Donor Characteristics of Extended Criteria Donor Cardiac Allografts, By Donor Sequence Number

Variable	Total *(n=12,438)*	DSN 1-5 *(n=8374)*	DSN 6-10 *(n=1537)*	DSN >10 *(n=2527)*	p value
Demographics					
Age, y	32 (24-42)	31 (23-40)	35 (26-43)	37 (28-47)	**<0.001**
Age ≥55 y	689 (5.54%)	342 (4.08%)	95 (6.18%)	252 (9.97%)	**<0.001**
BMI, kg/m^2^	27.3 (23.5-32.7)	27.0 (23.4-32.1)	27.8 (23.6-33.2)	28.3 (24.2-34.2)	**<0.001**
Sex mismatch	2854 (22.95%)	1765 (21.08%)	371 (24.14%)	718 (28.41%)	**<0.001**
Male donor-female recipient	1300 (10.45%)	945 (11.28%)	148 (9.63%)	207 (8.19%)	**<0.001**
Female donor-male recipient	1554 (12.49%)	820 (9.79%)	223 (14.51%)	511 (20.22%)	**<0.001**
Size mismatch	9493 (76.33%)	6515 (77.81%)	1148 (74.69%)	1830 (72.42%)	**<0.001**
Undersized donor (<80% recipient weight)	4075 (32.77%)	2893 (34.55%)	457 (29.73%)	725 (28.69%)	**<0.001**
Oversized donor (>120% recipient weight)	5418 (43.56%)	3622 (43.26%)	691 (44.96%)	1105 (43.73%)	**<0.001**
Medical history					
Diabetes	561 (4.55%)	315 (3.79%)	78 (5.11%)	168 (6.71%)	**<0.001**
Cancer	182 (1.48%)	107 (1.29%)	24 (1.57%)	51 (2.04%)	**0.022**
MI	113 (0.92%)	66 (0.79%)	17 (1.12%)	30 (1.20%)	0.199
Echocardiography					
LVEF, %	60 (55-65)	60 (56-65)	60 (55-65)	60 (55-65)	**<0.001**
LVEF <50%	383 (3.08%)	220 (2.63%)	50 (3.26%)	113 (4.48%)	**<0.001**
LV wall thickness >11 mm	2205 (17.73%)	1495 (17.85%)	276 (17.96%)	434 (17.17%)	0.713
Septal thickness >11 mm	2219 (17.84%)	1482 (17.70%)	273 (17.76%)	464 (18.36%)	0.744
Structural valvular abnormalities	55 (0.44%)	31 (0.37%)	5 (0.33%)	19 (0.75%)	**0.031**
Wall motion abnormalities	184 (1.48%)	106 (1.27%)	23 (1.50%)	55 (2.18%)	**0.004**
Coronary artery disease on catheterization	921 (7.40%)	478 (5.71%)	137 (8.91%)	306 (12.11%)	**<0.001**
Number ECD criteria met					**<0.001**
1	9614 (77.30%)	6602 (78.84%)	1182 (76.90%)	1830 (72.42%)	
2	2036 (16.37%)	1296 (15.48%)	258 (16.79%)	482 (19.07%)	
3	698 (5.61%)	433 (5.17%)	81 (5.27%)	184 (7.28%)	
4	83 (0.67%)	39 (0.47%)	15 (0.98%)	29 (1.15%)	
5	5 (0.04%)	4 (0.05%)	0 (0.00%)	1 (0.04%)	
6	2 (0.02%)	0 (0.00%)	1 (0.07%)	1 (0.04%)	
Multiple ECD criteria met	2824 (22.70%)	1772 (21.16%)	355 (23.10%)	697 (27.58%)	**<0.001**
Cause of death					
Cardiovascular	314 (2.52%)	187 (2.23%)	47 (3.06%)	80 (3.17%)	**0.012**
Stroke	2355 (18.93%)	1420 (16.96%)	314 (20.43%)	621 (24.57%)	**<0.001**
Trauma	5120 (41.16%)	3908 (46.67%)	536 (34.87%)	676 (26.75%)	**<0.001**
Cardiac arrest post-brain death	931 (7.64%)	595 (7.25%)	114 (7.64%)	222 (8.97%)	**0.019**
Blood type					**<0.001**
A	4528 (36.40%)	3139 (37.49%)	567 (36.89%)	822 (32.53%)	
AB	139 (1.12%)	124 (1.48%)	11 (0.72%)	4 (0.16%)	
B	1330 (10.69%)	982 (11.73%)	145 (9.43%)	203 (8.03%)	
O	6441 (51.78%)	4129 (49.31%)	814 (52.96%)	1498 (59.28%)	
Creatinine, mg/dL	1.0 (0.8-1.5)	1.0 (0.7-1.7)	1.0 (0.7-1.7)	1.0 (0.7-1.7)	0.316

Categorical data are expressed as n (%) and continuous data as medians (interquartile range). Bold type denotes p<0.05. BMI, body mass index; DSN, donor sequence number; ECD, extended criteria donor; MI, myocardial infarction; LV, left ventricle; LVEF, left ventricular ejection fraction.

#### Recipient characteristics

Recipients with a lower DSN were slightly younger, but not to a clinically significant degree (Table 2). Those with DSN >10 were more likely to be female and of white race than the DSN 1–5 group (Table 2). Compared to those with DSN 1–5, recipients with DSN >10 were less likely to be of high listing status (1A in the pre-October 2018 allocation era or 1–2 in the new era) and had longer waitlist times (Table 2). Pre-transplant intensive care unit admission and mechanical circulatory support were all less frequent in the DSN >10 group, compared to DSN 1–5 (Table 2).

**Table 2:** Baseline Recipient Characteristics of Extended Criteria Donor Heart Transplants at Time of Transplant, by Donor Sequence Number

Variable	Total *(n=12,438)*	DSN 1-5 *(n=8374)*	DSN 6-10 *(n=1537)*	DSN >10 *(n=2527)*	p value
Demographics					
Age, y	57 (47-64)	56 (46-63)	57 (47-64)	58 (49-65)	**<0.001**
Female sex	3388 (27.24%)	2013 (24.04%)	485 (31.55%)	890 (35.22%)	**<0.001**
Non-white race	4629 (37.22%)	3182 (38.00%)	586 (38.13%)	861 (34.07%)	**0.001**
BMI, kg/m^2^	27.4 (23.5-31.8)	27.4 (23.5-31.8)	27.3 (23.5-31.5)	27.5 (23.7-31.7)	0.340
Cardiomyopathy					**0.005**
Ischemic	3872 (31.13%)	2576 (30.76%)	480 (31.23%)	816 (32.29%)	
Restrictive	536 (4.31%)	337 (4.02%)	79 (5.14%)	120 (4.75%)	
Hypertrophic	360 (2.89%)	216 (2.58%)	56 (3.64%)	88 (3.48%)	
Dilated	6723 (54.05%)	4589 (54.80%)	800 (52.05%)	1334 (52.79%)	
Other	947 (7.61%)	656 (7.83%)	122 (7.94%)	169 (6.69%)	
Comorbidities					
Diabetes	3540 (28.48%)	2397 (28.64%)	438 (28.52%)	705 (27.91%)	0.773
Dialysis	284 (2.28%)	222 (2.65%)	22 (1.43%)	40 (1.58%)	**<0.001**
Smoking history	5487 (44.11%)	3712 (44.33%)	683 (44.44%)	1092 (43.21%)	0.591
Cerebrovascular disease	871 (7.05%)	580 (6.97%)	111 (7.27%)	180 (7.17%)	0.883
Prior cardiac surgery	5941 (48.51%)	4030 (48.84%)	707 (46.67%)	1204 (48.51%)	0.297
Severe functional impairment	5562 (44.72%)	4090 (48.84%)	702 (45.67%)	770 (30.47%)	**<0.001**
Status- before 10/18/2018					**<0.001**
1A	4530 (66.61%)	3458 (70.93%)	414 (59.91%)	658 (53.28%)	
1B	2011 (29.57%)	1333 (27.34%)	244 (35.31%)	434 (35.14%)	
2	260 (3.82%)	84 (1.72%)	33 (4.78%)	143 (11.58%)	
Status- after 10/18/2018					**<0.001**
1	555 (9.85%)	526 (15.03%)	19 (2.25%)	10 (0.77%)	
2	2669 (47.35%)	1975 (56.44%)	431 (50.95%)	263 (20.36%)	
3	1011 (17.94%)	539 (15.40%)	170 (20.09%)	302 (23.37%)	
4	1076 (19.09%)	354 (10.12%)	181 (21.39%)	541 (41.87%)	
5	4 (0.07%)	1 (0.03%)	2 (0.24%)	1 (0.08%)	
6	322 (5.71%)	104 (2.97%)	43 (5.08%)	175 (13.54%)	
Blood type					**<0.001**
A	4934 (39.67%)	3334 (39.81%)	629 (40.92%)	971 (38.43%)	
AB	663 (5.33%)	519 (6.20%)	75 (4.88%)	69 (2.73%)	
B	1873 (15.06%)	1342 (16.03%)	211 (13.73%)	320 (12.66%)	
O	4968 (39.94%)	3179 (37.96%)	622 (40.47%)	1167 (46.18%)	
Waitlist support					
Life support	10560 (84.90%)	7359 (87.88%)	1280 (83.28%)	1921 (76.02%)	**<0.001**
Inotropes	5918 (47.58%)	4144 (49.49%)	718 (46.71%)	1056 (41.79%)	**<0.001**
IABP	2386 (19.18%)	1730 (20.66%)	350 (22.77%)	306 (12.11%)	**<0.001**
ECMO	455 (3.66%)	400 (4.78%)	24 (1.56%)	31 (1.23%)	**<0.001**
Mechanical ventilation	2292 (18.43%)	1650 (19.70%)	228 (14.83%)	414 (16.38%)	**<0.001**
Waiting list time, d	61 (15-235)	60 (15-235)	47 (13-201)	72 (17-257)	**<0.001**
Transplant year	2018 (2015-2020)	2018 (2015-2020)	2019 (2016-2021)	2018 (2015-2021)	**<0.001**
Center ECD HT volume year of transplant, cases/year	14 (8-22)	17 (11-25)	19 (11-28)	15 (9-24)	**<0.001**
Low (1-7)	2409 (19.37%)	1884 (22.50%)	212 (13.79%)	313 (12.39%)	
Medium (8-15)	3851 (30.96%)	2714 (32.41%)	464 (30.19%)	673 (26.63%)	
High (>15)	6178 (49.67%)	3776 (45.09%)	861 (56.02%)	1541 (60.98%)	
Days admitted before transplant	4 (1-23)	7 (1-26)	4 (1-20)	1 (1-13)	**<0.001**
ICU pre-transplant	5098 (40.99%)	3757 (44.87%)	643 (41.83%)	698 (27.62%)	**<0.001**
Lab data					
Creatinine, mg/dL	1.2 (0.9-1.4)	1.2 (0.9-1.4)	1.1 (0.9-1.4)	1.2 (0.9-1.4)	0.913
Total bilirubin, mg/dL	0.7 (0.5-1.1)	0.7 (0.5-1.1)	0.7 (0.5-1.1)	0.6 (0.4-1.0)	**<0.001**
Hemodynamic data					
Cardiac index, L/min m^2^	2.2 (1.8-2.6)	2.2 (1.8-2.6)	2.1 (1.7-2.6)	2.2 (1.8-2.6)	**0.009**
Mean PAP, mmHg	26 (19-34)	27 (20-34)	26 (19-34)	24 (18-32)	**<0.001**
PCWP, mmHg	17 (11-24)	17 (11-24)	17 (10-24)	15 (10-22)	**<0.001**
PVR, WU	2.0 (1.3-3.0)	2.0 (1.3-3.0)	2.0 (1.3-3.0)	2.0 (1.3-2.9)	0.381

Categorical data are expressed as n (%) and continuous data as medians (interquartile range). Bold type denotes p<0.05. BMI, body mass index; DSN, donor sequence number; IABP, intra-aortic balloon pump; ECD, extended criteria donor; ECMO, extracorporeal membrane oxygenation; HT, heart transplant; ICU, intensive care unit; PAP, pulmonary artery pressure; PCWP, pulmonary capillary wedge pressure; PVR, pulmonary vascular resistance. Smoking history was positive if ≥10 pack-year history. Severe functional impairment defined as ≤40% on Karnofsky performance scale. Old and new allocation systems refer to change in United States donor heart allocation policy on October 18, 2018. Cutoffs for low, medium, and high center volume were determined by tertiles of ECD HTs performed in 2022.

#### Transplant characteristics and post-transplant outcomes

**Figure 3:**
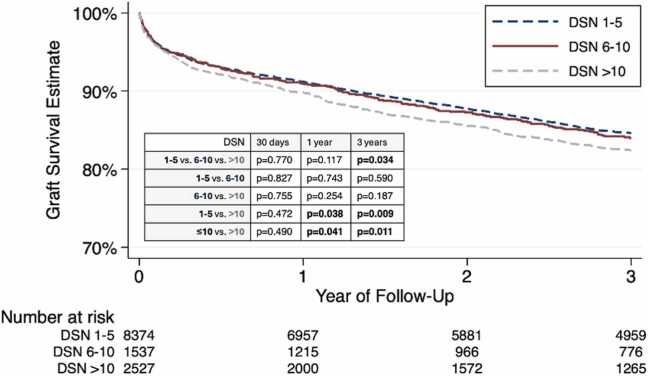
3-year Kaplan-Meier graft survival estimates after extended criteria donor heart transplant, by donor sequence number (DSN). Log-rank p value shown. Bold type denotes p<0.05.

Ischemic time and donor distance both increased with DSN (Table 3). Post-operative length of stay and most in-hospital adverse outcomes (except acute rejection before discharge, which was more common in the DSN>10 group) were similar across cohorts (Table 3). 30-day mortality was 3.3% overall and did not differ by cohort (Table 3). By 1 year post-transplant, graft survival estimates were significantly lower in patients whose DSN was >10, compared to ≤10 (89.8% vs. 91.1%, log-rank p=0.041, Figure 3). This effect persisted at 3 years post-transplant, at which point graft survival in the DSN >10 group was 82.4%, compared to 84.5% in the ≤10 offers cohort (log-rank p=0.011, Figure 3). Receipt of an ECD heart that had been offered >10 times prior to acceptance was an independent risk factor for 3-year graft failure on both univariable (hazard ratio 1.16 [1.03–1.29], p=0.011) and multivariable (adjusted hazard ratio 1.13 [1.01–1.27], p=0.039) Cox regression (Table 4, Figure 5), but not multivariate-adjusted hazard of 30-day or 1-year graft failure (Table S3). Subgroup analysis was performed for each individual ECD criteria, as well as prolonged ischemic time >4 h (Figure S3). Only in the LVEF <50% cohort was DSN >10 significantly associated with inferior 3-year graft survival (79.8% vs. 88.0%, log-rank p=0.035), compared to DSN ≤10.

**Table 3:** Transplant Characteristics and Post-transplant Outcomes of Extended Criteria Donor Heart Transplants, by Donor Sequence Number

Variable	Total *(n=12,438)*	DSN 1-5 *(n=8374)*	DSN 6-10 *(n=1537)*	DSN >10 *(n=2527)*	p value
Ischemic time, h	3.3 (2.6-3.9)	3.1 (2.4-3.8)	3.4 (2.9-4.0)	3.6 (3.0-4.2)	**<0.001**
Ischemic time >4 h	2748 (22.16%)	1514 (18.14%)	398 (25.96%)	836 (33.19%)	**<0.001**
Donor distance, miles	156 (28-350)	105 (16-297)	216 (78-399)	282 (117-464)	**<0.001**
Post-operative length of stay, d	16 (11-24)	16 (11-24)	16 (12-24)	16 (11-24)	1.000
Events prior to discharge					
Acute rejection	2304 (18.52%)	1488 (17.77%)	299 (19.45%)	517 (20.46%)	**0.006**
Dialysis	1621 (13.09%)	1086 (13.04%)	200 (13.05%)	335 (13.29%)	0.945
Stroke	425 (3.44%)	305 (3.67%)	44 (2.88%)	76 (3.02%)	0.132
Permanent pacemaker	297 (2.40%)	191 (2.29%)	34 (2.22%)	72 (2.86%)	0.228
Follow-up time, y	3.7 (1.2-6.1)	4.0 (1.8-6.7)	3.0 (1.0-5.8)	3.0 (1.0-6.0)	**<0.001**
Rejection within 1 year	1810 (17.35%)	1207 (17.09%)	237 (18.41%)	366 (17.57%)	0.492
Mortality					
30-day	409 (3.29%)	273 (3.26%)	52 (3.38%)	84 (3.32%)	0.963
1-year	1087 (8.74%)	709 (8.47%)	135 (8.78%)	243 (9.62%)	0.200
Cause of death					**0.045**
Graft failure	257 (10.80%)	177 (10.85%)	25 (9.06%)	55 (11.65%)	
Infection	397 (16.68%)	264 (16.18%)	43 (15.58%)	90 (19.07%)	
Cardiovascular	431 (18.11%)	298 (18.26%)	47 (17.03%)	86 (18.22%)	
Pulmonary	195 (8.19%)	124 (7.60%)	24 (8.70%)	47 (9.96%)	
Cerebrovascular	147 (6.18%)	88 (5.39%)	19 (6.88%)	40 (8.47%)	
Hemorrhage	56 (2.35%)	37 (2.27%)	7 (2.54%)	12 (2.54%)	
Malignancy	222 (9.33%)	158 (9.68%)	34 (12.32%)	30 (6.36%)	
Other	675 (28.36%)	486 (29.78%)	77 (27.90%)	112 (23.73%)	
Repeat transplant	118 (0.96%)	71 (0.85%)	16 (1.05%)	31 (1.24%)	0.211
Time to retransplant, y	2.9 (0.0-5.5)	2.6 (0.4-5.5)	4.8 (3.4-5.8)	2.6 (0.0-4.8)	0.090

Categorical data are expressed as n (%) and continuous data as medians (interquartile range). Bold type denotes p<0.05. DSN, donor sequence number.

**Table 4:** Cox Proportional-hazards Model for 3-year Graft Failure in Recipients of Extended Criteria Donor Hearts

Variable	Univariable Analysis		Multivariable Analysis	
	Hazard Ratio [95% CI]	p value	Adjusted Hazard Ratio [95% CI]	p value
DSN >10 (vs. ≤10)	1.16 [1.03-1.29]	**0.011**	1.13 [1.01-1.27]	**0.039**
Age >60 years	1.21 [1.10-1.33]	**<0.001**	1.21 [1.09-1.33]	**<0.001**
Non-white race	1.17 [1.06-1.28]	**0.001**	1.20 [1.09-1.32]	**<0.001**
BMI, kg/m^2^ (increasing)	1.02 [1.02-1.03]	**<0.001**	1.02 [1.01-1.03]	**<0.001**
Diabetes	1.28 [1.16-1.41]	**<0.001**	1.14 [1.03-1.27]	**0.010**
Dialysis	2.21 [1.76-2.77]	**<0.001**	2.02 [1.61-2.54]	**<0.001**
Smoking history	1.19 [1.08-1.30]	**<0.001**	1.17 [1.06-1.29]	**0.001**
Prior cardiac surgery	1.38 [1.26-1.52]	**<0.001**	1.34 [1.22-1.48]	**<0.001**
Creatinine ≥2.0 mg/dL	1.87 [1.58-2.20]	**<0.001**	1.62 [1.36-1.91]	**<0.001**
Total bilirubin ≥3.0 mg/dL	2.23 [1.84-2.71]	**<0.001**	2.13 [1.75-2.60]	**<0.001**
PVR >3 WU	1.18 [1.07-1.30]	**0.001**	1.23 [1.11-1.36]	**<0.001**
Days on waiting list (increasing)	1.00 [1.00-1.00]	**0.011**	1.00 [1.00-1.00]	0.078
New allocation era (≥10/18/2018)	1.11 [1.01-1.22]	**0.031**	1.14 [1.04-1.26]	**0.007**
Donor age ≥55 years	1.40 [1.17-1.67]	**<0.001**	1.27 [1.06-1.52]	**0.010**
Donor death due to stroke	1.20 [1.08-1.34]	**0.001**	1.18 [1.05-1.33]	**0.004**
Ischemic time >6 h	1.46 [1.11-1.92]	**0.007**	1.43 [1.09-1.89]	**0.010**

Univariable and multivariable hazard ratios with 95% confidence interval (CI) and p-value for interaction were calculated for association with 3-year graft failure. Bold type denotes p<0.05. BMI, body mass index; DSN, donor sequence number; PVR, pulmonary vascular resistance; WU, Wood units. Log likelihood ratio for the multivariable Cox proportional-hazards model is −16,085.112.

**Figure 4:**
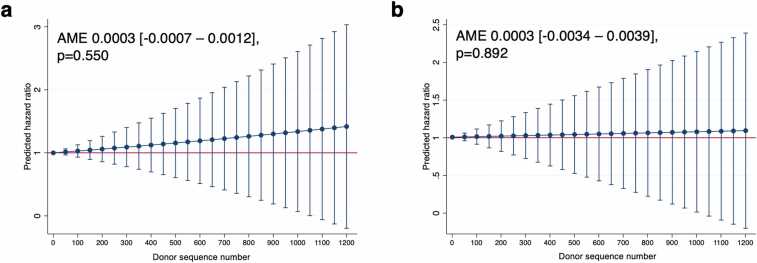
Unadjusted (a) and adjusted (b) predictive margins with 95% confidence intervals of donor sequence number on hazard of 3-year recipient graft failure in extended criteria donor heart transplant. Average marginal effect (AME) with 95% confidence intervals shown. The following adjustor variables were included in the multivariable model: recipient age >60 years, non-white race, body mass index (increasing), diabetes, dialysis, smoking history, prior cardiac surgery, creatinine ≥2.0 mg/dL, total bilirubin ≥3.0 mg/dL, pulmonary vascular resistance >3 Wood units, days on wait list (increasing), transplant in new allocation era (≥10/18/2018), donor age ≥55 years, donor death due to stroke, and ischemic time >6 h.

A single-unit increase in DSN did not significantly affect predicted hazard of 3-year graft failure in unadjusted (average marginal effect 0.0003 [−0.0007 – 0.0012], p=0.550) or adjusted (average marginal effect 0.0003 [−0.0034 – 0.0039], p=0.892) predictive margins calculated from the Cox regression models (Figure 4). A sensitivity analysis including center ECD HT volume as one of the multivariate adjustor variables yielded similar results (Table S4, Figure S4).

**Figure 5:**
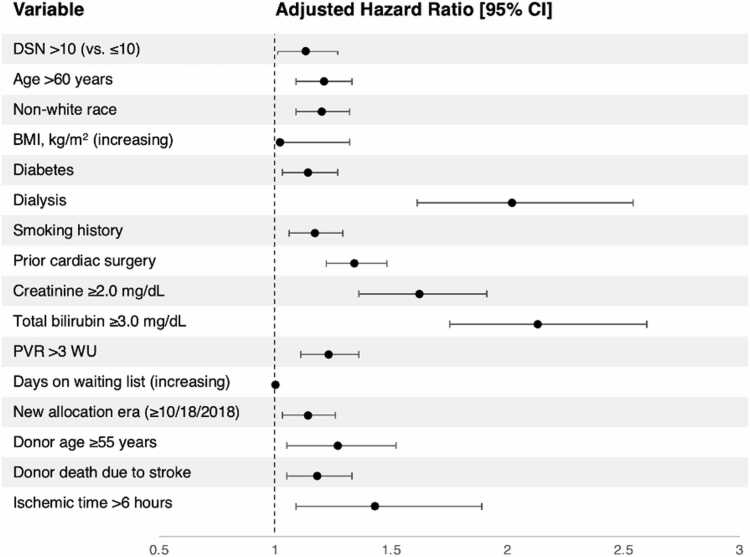
Multivariable Cox proportional-hazards model for 3-year graft failure in recipients of extended criteria donor hearts. Multivariable hazard ratios with 95% confidence interval (CI) and p-value for interaction were calculated for association with 3-year graft failure. Bold type denotes p<0.05. BMI, body mass index; DSN, donor sequence number; PVR, pulmonary vascular resistance; WU, Wood units.

## Discussion

Use of extended criteria donors is a growing strategy to bridge the allograft supply-demand gap in heart transplantation, but careful donor-recipient selection is imperative to ensure acceptable outcomes. Our analysis of the effect of DSN on post-transplant survival in ECD HT shows that the vast majority (80%) of ECD organs are accepted within 10 offers. Recipients with high DSN (>10) were more likely to be of lower medical acuity, were located farther from the donor, and underwent transplants with longer ischemic times. Despite lower-urgency recipient characteristics, higher DSN was associated with similar 30-day and slightly inferior 1-year and 3-year graft survival, compared to recipients with DSN ≤10. Importantly, this observed signal appears largely driven by donors with LVEF <50%, who represent a minority of the cohort. Additionally, incremental increases in DSN were associated with small, non-significant increases in risk of 3-year graft failure.

Previous work evaluating the significance of DSN in all HT recipients concluded that DSN is not independently associated with increased risk of post-transplant mortality and should not be relied on as a sole metric during the donor selection process. Baran et al. examined over 12,000 HTs between 2007–2014 using national data and observed no survival differences whether stratifying patients at various DSN cutoffs.^9^ Zhou et al. performed a more contemporary analysis of all US HT recipients between 2015–2022, categorizing recipients with the top 10th percentile of DSN at acceptance (≥42) as “high DSN.” They similarly identified no difference in short- or mid-term survival between the high and low DSN groups in both the overall cohort and a subgroup analysis of donation after circulatory death (DCD) transplants.^10^

In contrast, our study focuses on the unique subpopulation of ECD transplants. Can DSN reliably be used as a proxy for a lower quality allograft in this higher-risk population? In both our ECD cohort (Table 1) and that of Baran et al., higher DSN was indeed associated with higher incidence of most suboptimal donor characteristics, as well as donors that met multiple ECD criteria.^9^ Interestingly, we observed that weight-based size mismatch was the only non-standard criteria to be more common in the lower DSN groups than higher DSN (Table 1). One possible explanation for this finding is that transplant teams may be more willing to accept size mismatch than other high-risk features such as advanced donor age and signs of compromised cardiac function.

In addition to higher-risk donor features, outcomes after transplants performed using ECD allografts offered >10 times prior to acceptance were significantly inferior even after covariate adjustment compared to DSN ≤10 (Figure 3, Table 4). These results demonstrate that allocating high-DSN ECD organs to low-risk patients still lends a signal toward suboptimal outcomes, despite lower recipient medical urgency (Table 2). It was previously demonstrated that in lower-urgency (status 4 or 6) patients, receipt of an ECD heart (vs. a standard heart) did not impact survival (whereas ECD transplant was associated with worse survival in status 1 and 2 patients).^7^ This and other work showing that high-risk donor/low-risk recipient pairings lead to less graft failure than low-risk donor/high-risk recipient pairings have provided support for transplant teams’ increased willingness to accept marginal donors for lower-risk recipients.^13^ Indeed, the lower acuity of high-DSN recipients may also be explained by inherent lower priority of candidates who are positioned lower on the ranked allocation list. However, our findings may put into question the practice of accepting a marginal donor with many prior offers to accelerate transplant for a lower-acuity recipient who may otherwise have to wait a long time for an organ offer. In particular, high DSN in combination with donor LVEF <50% should merit careful deliberation by the transplant team, as this specific ECD characteristic was the only one resulting in significantly inferior post-transplant outcomes on subgroup analysis (Figure S3).

Although DSN >10 is evidently associated with inferior post-transplant outcomes, particularly in the case of depressed donor EF, analysis of DSN as a continuous variable is more revealing. Despite an overall trend pointing toward a positive association between DSN and rates of graft failure, single-unit increments in DSN were associated with nearly negligible increases in hazard of 3-year graft failure, with differences in DSN on the scale of hundreds needed to see a meaningful increase in risk (Figure 4). Overall, while our data demonstrates a signal for association between very high DSN and inferior post-transplant outcomes in the ECD population, small differences in DSN (i.e. 1 vs. 2, or even 50 vs. 51) are unlikely to lead to any clinically meaningful increase in risk. Rather than an independent risk factor, DSN should be interpreted judiciously in combination with all other donor and recipient characteristics.

The visibility of the DSN means that transplant teams may be subconsciously—or consciously—influenced by their perception of how colleagues at other centers have evaluated the same allograft. In an editorial, Farr and Colvin describe the use of DSN as a “middle of the night heuristic on which [transplant teams] rely to efficiently field offers,” likening this situation to groupthink.^14^ Groupthink is a form of systematic bias in group decision making in which premature consensus seeking impedes effective decision making.^15^ Interest in understanding how groupthink in healthcare settings may impact patient care is growing, but this phenomenon is difficult to assess objectively, with the majority of literature consisting of anecdotal examples rather than empirical studies.^15^ In the present case, the “group” is transplant teams at other centers that have previously evaluated and turned down the organ. If physicians anchor on the DSN or quickly align with the “group’s” consensus without critically evaluating all information, rational decision-making may be compromised.^16^ DSN is not a perfect correlate for organ quality, as organ offers may be rejected for recipient or logistic reasons that are unrelated to donor quality.^9^ Furthermore, DSN does not necessarily represent the number of transplant teams that have evaluated the allograft: if an offer is made to a transplant center for multiple recipients in sequence and that center turns down the organ for all of its candidates, the DSN can escalate rapidly, as it is a marker of each individual recipient for whom the organ has been declined.^9^ Similarly, the DSN could “freefall” if center after center rejects the organ in rapid crowd consensus.^14^ However, groupthink in donor selection is not unilaterally negative: in the setting of a high DSN and otherwise promising donor/recipient match, the DSN may prompt a mental check for the team to ensure they are not missing something important about the donor that led the previous centers to decline the allograft.^14^ Ultimately, transplant teams must be careful to avoid heuristic thinking when evaluating organ offers.

Although prolonged ischemic time is a major risk factor for primary graft dysfunction (PGD), we chose to not include it in our ECD definition as it is not strictly a donor characteristic and can be challenging to predict at time of donor selection, even though it is often related to donor distance.^1^ In our study, although higher DSN was associated with longer ischemic times, median ischemic time was <4 h even in the DSN >10 group (Table 3). Transplant teams may be more willing to accept perceived lower-quality allografts (i.e. ECD, higher DSN) if they anticipate that they will be able to accomplish the transplant with a shorter ischemic time. Heart transplant practice patterns are rapidly evolving. Advances made during and since our study period of 2012–2022, including DCD, normothermic regional perfusion, and organ preservation technologies, may limit the applicability of our findings to current practice. In the future, wider adoption of organ preservation technologies such as the Organ Care System (TransMedics) will help limit cold ischemic time, avoid PGD, and broaden utilization of ECD allografts.^17^ Further work is needed to better understand the interaction of ECD, DCD and high DSN in risk prognostication and donor selection.

In addition to the above, other relevant limitations affect this study. Like any retrospective analysis of the UNOS database, lack of clinical granularity and incomplete data reporting limited our ability to assess some key variables, including PGD, panel reactive antibodies, and organ preservation technologies. Although we based our ECD definition upon 2020 ISHLT recommendations on donor selection, there remains no standard consensus on the set of characteristics that should be characterized as ECD. While the 2020 recommendations include weight-based size mismatch as a parameter, predicted heart mass (PHM) is increasingly recognized as the optimal metric for assessing size compatibility; mortality risk may be lower when classifying by PHM.^18^, ^19^ In addition, donor-recipient matching is highly individualized and we are unable to account for human bias and differences in expertise inherent to the selection process. We also fail to capture a “denominator”, and do not analyze hearts that were ultimately rejected.

In conclusion, while previous work demonstrated that DSN does not impact outcomes in the overall HT population, our analysis suggests that use of an extended criteria donor allograft with many rejections prior to acceptance may be associated with suboptimal post-transplant graft survival. However, large margins in DSN are needed to observe meaningful increase in hazard of graft failure. Additionally, the association between DSN >10 and lower 3-year graft survival was largely driven by the subset of donors with LVEF <50% who comprised only 3% of all donors. Although transplant teams should be cognizant to not anchor on and decline organs solely based on DSN, it may serve as a useful tool when evaluating ECD organ offers.

## CRediT authorship contribution statement

C.S., D.R., A.I., and M.C. conceived the study design. C.S., D.R., and M.A. performed the data analysis. C.S. wrote the manuscript with support from A.I., M.S., N.W., O.T., M.B., and M.C. All authors discussed the results and contributed to the final manuscript.

## Financial disclosure statement

None of the authors of this paper report any financial conflicts of interest or sources of funding relevant to this research.

## Declaration of Competing Interest

The authors declare that they have no known competing financial interests or personal relationships that could have appeared to influence the work reported in this paper.

## Footnotes

Appendix A Supplemental data associated with this article can be found in the online version at doi:10.1016/j.jhlto.2026.100566.
