# Supplementary material for: Use of extended criteria donor cardiac allografts after multiple offers is associated with inferior post-transplant outcomes
Source: JHLT Open. 2026 Apr 16;13:100566. doi: 10.1016/j.jhlto.2026.100566 (PMC13157042; doi:10.1016/j.jhlto.2026.100566)
Supplement: Supplementary file 1 — Supplemental material [file mmc1.docx]

**SUPPLEMENTAL MATERIAL**

**Figure S1.** Restricted cubic spline of donor sequence number and 3-year graft failure hazard ratio.

Predicted hazard ratios for 3-year graft failure were calculated and anchored at donor sequence number (DSN) = 1. Slopes of hazard ratio curve were calculated across the observed DSN region and noted to change substantially in the region of DSN 4-7, identifying an inflection region.

**Figure S2.** Frequency of individual extended criteria donor criteria.

**
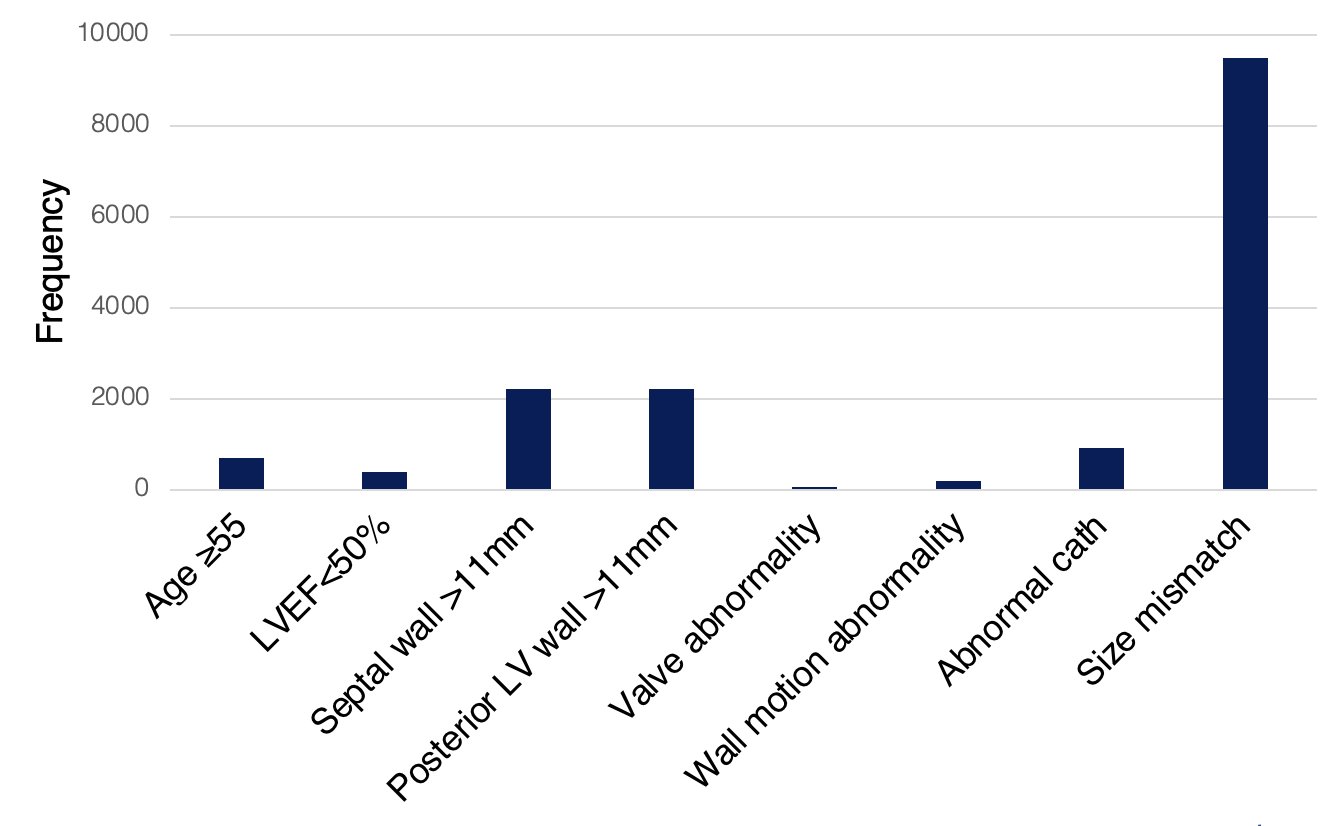
**

LV, left ventricular; LVEF, left ventricular ejection fraction.

**Figure S3.** 3-year Kaplan-Meier graft survival estimates after extended criteria donor heart transplant, by donor sequence number (DSN), in subgroups corresponding to each individual extended criteria donation criterion (a-h) as well as ischemic time >4 hours (i).

**
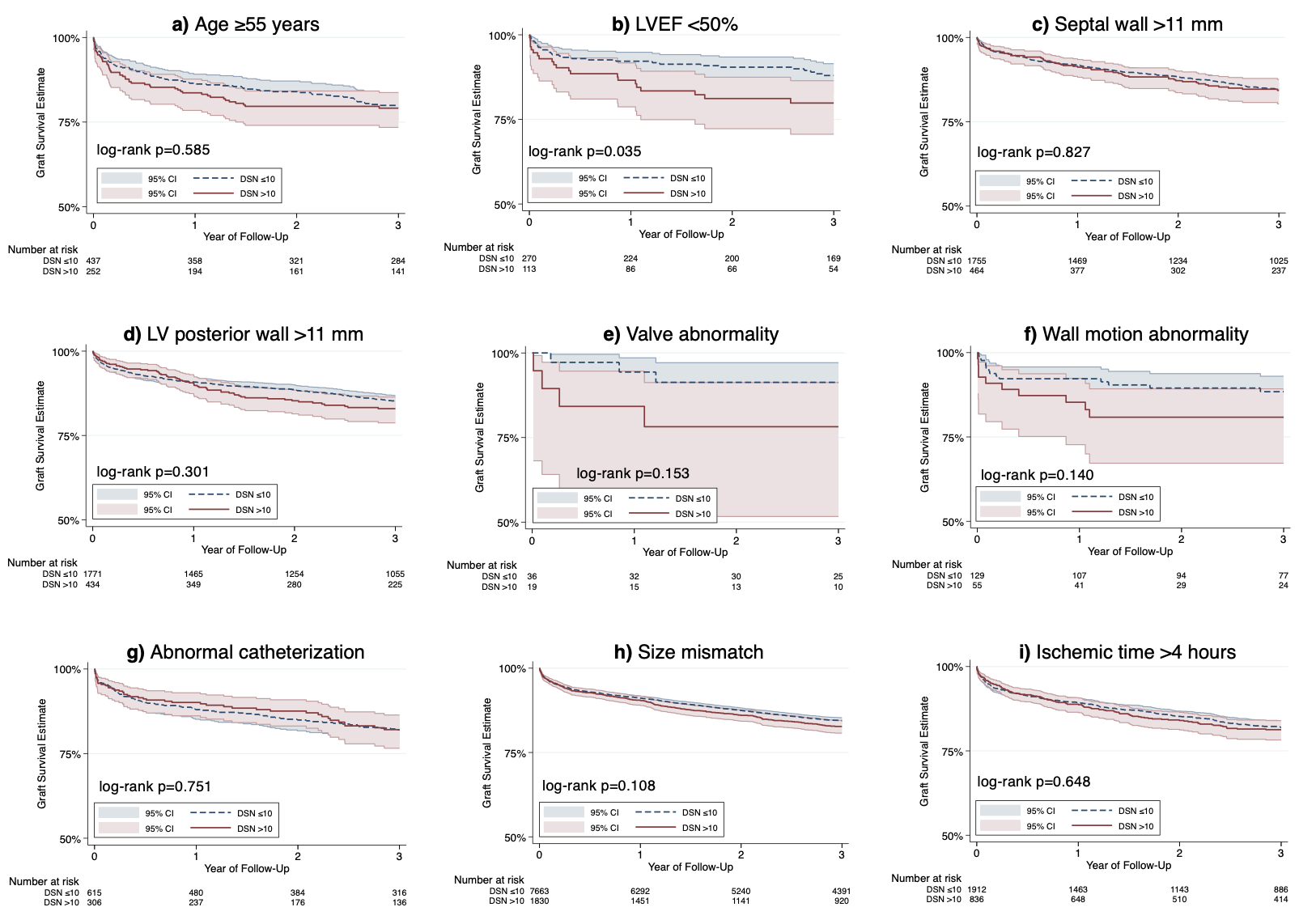
**

95% confidence intervals shown.

**Figure S4.** Adjusted predictive margins with 95% confidence intervals of donor sequence number on hazard of 3-year recipient graft failure in extended criteria donor heart transplant, including center volume as an adjustor variable.

Average marginal effect (AME) with 95% confidence intervals shown. The following adjustor variables were included in the multivariable model: recipient age >60 years, non-white race, body mass index (increasing), diabetes, dialysis, smoking history, prior cardiac surgery, creatinine ≥2.0 mg/dL, total bilirubin ≥3.0 mg/dL, pulmonary vascular resistance >3 Wood units, days on wait list (increasing), transplant in new allocation era (≥10/18/2018), donor age ≥55 years, donor death due to stroke, ischemic time >6 hours, and annual center extended criteria donor heart transplant volume (increasing).

**Table S1.** Variables with non-zero missingness.

| **Variable** | **Missingness** |
| --- | --- |
| **Recipient characteristics at time of transplant** |  |
| **Age, y** | 0.01%, n=1 |
| **BMI, kg/m^2^** | 0.05%, n=7 |
| **Diabetes** | 0.06%, n=8 |
| **Cerebrovascular disease** | 0.67%, n=83 |
| **Prior cardiac surgery** | 1.53%, n=190 |
| **Status- after 10/18/2018** | 0.60%, n=34 |
| **Total bilirubin, mg/dL** | 0.15%, n=19 |
| **Cardiac index, L/min m^2^** | 3.9%, n=488 |
| **Mean PAP, mmHg** | 2.3%, n=281 |
| **PCWP, mmHg** | 7.0%, n=874 |
| **PVR, WU** | 8.6%, n=1074 |
|  |  |
| **Transplant characteristics** |  |
| **Ischemic time, h** | 0.3%, n=38 |
|  |  |
| **Donor characteristics** |  |
| **BMI, kg/m^2^** | 0.0%, n=1 |
| **Size mismatch** | 0.0%, n=1 |
| **Diabetes** | 0.8%, n=102 |
| **Cancer** | 0.8%, n=101 |
| **Myocardial infarction** | 0.9%, n=113 |
| **LVEF, %** | 0.0%, n=7 |
| **LVEF <50%** | 0.0%, n=7 |
| **LV wall thickness >11mm** | 30.5%, n=3796 |
| **Septal thickness >11 mm** | 33.3%, n=4147 |
| **Structural valvular abnormalities** | 96.9%, n=12055 |
| **Wall motion abnormalities** | 96.9%, n=12055 |
| **Coronary artery disease on catheterization** | 56.7%, n=7051 |
| **Cardiac arrest post-brain death** | 2.1%, n=260 |
|  |  |
| **Post-transplant outcomes** |  |
| **Post-operative length of stay, d** | 1.1%, n=131 |
| **Dialysis** | 0.5%, n=56 |
| **Stroke** | 0.6%, n=75 |
| **Permanent pacemaker** | 0.3%, n=39 |
| **Rejection within 1 year** | 16.1%, n=2006 |
| **Cause of death** | 14.2%, n=394 |
| **Repeat transplant** | 0.75%, n=93 |

BMI, body mass index; LV, left ventricle; LVEF, left ventricular ejection fraction; PAP, pulmonary artery pressure; PCWP, pulmonary capillary wedge pressure; PVR, pulmonary vascular resistance; WU, Wood units.

**Table S2.** Univariable Cox proportional-hazard ratios for 3-year graft failure in recipients of ECD hearts.

| **Variable** | **Hazard Ratio [95% CI]** | **p value** |
| --- | --- | --- |
| **DSN (increasing)** | 1.00 [1.00-1.00] | 0.548 |
| **DSN 6-10 vs. DSN 1-5** | 1.04 [0.90-1.20] | 0.592 |
| **DSN >10 vs. DSN 1-5** | 1.16 [1.04-1.30] | **0.009** |
| **DSN >10 vs. DSN 6-10** | 1.12 [0.95-1.32] | 0.187 |
| **DSN >10 vs. DSN ≤10** | 1.16 [1.03-1.29] | **0.011** |
|  |  |  |
| ***Recipient characteristics at transplant*** |  |  |
| **Age >60 years** | 1.21 [1.10-1.33] | **<0.001** |
| **Female sex** | 0.99 [0.89-1.10] | 0.891 |
| **Non-white race** | 1.17 [1.06-1.28] | **0.001** |
| **BMI, kg/m^2^ (increasing)** | 1.02 [1.02-1.03] | **<0.001** |
| **Diabetes** | 1.28 [1.16-1.41] | **<0.001** |
| **Dialysis** | 2.21 [1.76-2.77] | **<0.001** |
| **Smoking history** | 1.19 [1.08-1.30] | **<0.001** |
| **Prior cardiac surgery** | 1.38 [1.26-1.52] | **<0.001** |
| **Severe functional impairment** | 1.02 [0.93-1.12] | 0.705 |
| **Status 1A or 1-2** | 0.95 [0.87-1.05] | 0.338 |
| **Blood type** | 1.01 [1.00-1.03] | 0.078 |
| **Days on waiting list (increasing)** | 1.00 [1.00-1.00] | **0.011** |
| **Transplant year (increasing)** | 1.01 [0.99-1.02] | 0.356 |
| **New allocation era (transplanted on/after 10/18/2018)** | 1.11 [1.01-1.22] | **0.031** |
| **Days admitted prior to transplant (increasing)** | 1.00 [1.00-1.00] | 0.985 |
| **Any IABP** | 0.91 [0.80-1.03] | 0.130 |
| **Any inotropes** | 0.92 [0.84-1.01] | 0.084 |
| **Any mechanical ventilation** | 1.18 [1.06-1.33] | **0.004** |
| **ICU prior to transplant** | 0.96 [0.88-1.06] | 0.443 |
| **Creatinine ≥2.0 mg/dL** | 1.87 [1.58-2.20] | **<0.001** |
| **Total bilirubin ≥3.0 mg/dL** | 2.23 [1.84-2.71] | **<0.001** |
| **Cardiac index <2.2 L/min/m^2^** | 1.03 [0.94-1.13] | 0.560 |
| **Mean PAP >30 mmHg** | 1.02 [0.93-1.12] | 0.742 |
| **PCWP >15 mmHg** | 0.94 [0.86-1.04] | 0.232 |
| **PVR >3 WU** | 1.18 [1.07-1.30] | **0.001** |
|  |  |  |
| ***Donor and transplant characteristics*** |  |  |
| **Donor age ≥55** | 1.40 [1.17-1.67] | **<0.001** |
| **Donor BMI, kg/m^2^ (increasing)** | 1.00 [0.99-1.00] | 0.378 |
| **Sex mismatch** | 1.06 [0.95-1.18] | 0.309 |
| **Weight-based size mismatch >20%** | 0.99 [0.89-1.11] | 0.893 |
| **LVEF<50%** | 0.93 [0.70-1.22] | 0.594 |
| **LV wall thickness>11mm** | 0.96 [0.85-1.10] | 0.563 |
| **Septal thickness>11mm** | 0.95 [0.84-1.08] | 0.458 |
| **Structural valvular abnormality** | 0.91 [0.41-2.03] | 0.827 |
| **Wall motion abnormality** | 1.00 [0.58-1.73] | 0.996 |
| **Abnormal cardiac catheterization** | 1.12 [0.94-1.35] | 0.201 |
| **Donor death due to stroke** | 1.20 [1.08-1.34] | **0.001** |
| **Donor death due to trauma** | 1.01 [0.92-1.11] | 0.832 |
| **Cardiac arrest post-brain death** | 0.90 [0.74-1.08] | 0.241 |
| **Donor blood type** | 1.01 [1.00-1.03] | 0.097 |
| **Donor creatinine ≥2.0 mg/dL** | 0.97 [0.86-1.09] | 0.616 |
| **Ischemic time >6 hours** | 1.46 [1.11-1.92] | **0.007** |
| **Donor distance, miles (increasing)** | 1.00 [1.00-1.00] | 0.255 |
| **Donor distance >250 miles** | 1.04 [0.95-1.15] | 0.380 |
| **Donor distance >500 miles** | 1.03 [0.87-1.21] | 0.750 |
| **Center ECD HT volume (cases/year, increasing)** | 0.94 [0.89-1.00] | 0.061 |
| **High center ECD HT volume (>15 cases/year)** | 0.95 [0.87-1.05] | 0.325 |

Univariable hazard ratios for graft failure with 95% confidence interval (CI) and p-value for interaction were calculated for association with 3-year graft failure. Bold type denotes p<0.05. BMI, body mass index; DSN, donor sequence number; ECD, extended criteria donor; HT, heart transplant; IABP, intra-aortic balloon pump; ICU, intensive care unit; LV, left ventricular; LVEF, left ventricular ejection fraction; PAP, pulmonary artery pressure; PCWP, pulmonary capillary wedge pressure; PVR, pulmonary vascular resistance; WU, Wood units.

**Table S3.** Cox proportional-hazards models for 30-day and 1-year graft failure in recipients of extended criteria donor hearts.

| **Variable** | **30-day graft failure** | | **1 year graft failure** | |
| --- | --- | --- | --- | --- |
|  | **Adjusted Hazard Ratio [95% CI]** | **p value** | **Adjusted Hazard Ratio [95% CI]** | **p value** |
| **DSN >10 (vs. ≤10)** | 1.09 [0.86-1.37] | 0.492 | 1.12 [0.97-1.30] | 0.114 |
| **Age >60 years** | 1.12 [0.92-1.37] | 0.266 | 1.34 [1.18-1.51] | **<0.001** |
| **Non-white race** | 0.87 [0.71-1.07] | 0.188 | **1.00 [0.88-1.14]** | 0.967 |
| **BMI, kg/m^2^ (increasing)** | 1.06 [1.04-1.08] | **<0.001** | 1.03 [1.01-1.04] | **<0.001** |
| **Diabetes** | 0.89 -0.72-1.09] | 0.255 | 1.16 [1.02-1.32] | **0.024** |
| **Dialysis** | 2.44 [1.64-3.63] | **<0.001** | 2.37 1.83-3.09] | **<0.001** |
| **Smoking history** | 1.30 [1.08-1.58] | **0.007** | 1.14 [1.01-1.28] | **0.035** |
| **Prior cardiac surgery** | 1.85 [1.51-2.26] | **<0.001** | 1.44 [1.27-1.63] | **<0.001** |
| **Creatinine ≥2.0 mg/dL** | 1.75 [1.27-2.40] | **0.001** | 1.81 [1.48-2.21] | **<0.001** |
| **Total bilirubin ≥3.0 mg/dL** | 3.13 [2.26-4.33] | **<0.001** | 2.57 [2.05-3.23] | **<0.001** |
| **PVR >3 WU** | 1.36 [1.11-1.67] | **0.003** | 1.30 [1.15-1.48] | **<0.001** |
| **Days on waiting list (increasing)** | 1.00 [1.00-1.00] | 0.836 | 1.00 [1.00-1.00] | 0.005 |
| **New allocation era (≥10/18/2018)** | 0.89 [0.73-1.09] | 0.256 | 1.08 [0.96-1.22] | 0.218 |
| **Donor age ≥55 years** | 1.47 [1.04-2.07] | **0.030** | 1.50 [2.22-1.86] | **<0.001** |
| **Donor death due to stroke** | 1.24 [0.98-1.56] | 0.071 | 1.23 [1.07-1.42] | **0.004** |
| **Ischemic time >6 hours** | 2.02 [1.28-3.18] | **0.003** | 1.45 [1.04-2.03] | **0.030** |
| **Center ECD HT volume (cases/year, increasing)** | 1.00 [0.99-1.00] | 0.412 | 1.00 [0.99-1.00] | 0.191 |

Multivariable hazard ratios with 95% confidence interval (CI) and p-value for interaction were calculated for association with 30-day and 1-year graft failure. Bold type denotes p<0.05. BMI, body mass index; DSN, donor sequence number; ECD, extended criteria donor; HT, heart transplant; PVR, pulmonary vascular resistance; WU, Wood units. Log likelihood ratios for the multivariable Cox proportional-hazards model are -3989.84 for 30-day graft failure and -10207.14 for 1-year graft failure.

**Table S4.** Multivariable Cox proportional-hazards model for 3-year graft failure in recipients of extended criteria donor hearts, including center volume.

| **Variable** | **MULTIVARIABLE ANALYSIS** | |
| --- | --- | --- |
|  | **Adjusted Hazard Ratio [95% CI]** | **p value** |
| **DSN >10 (vs. ≤10)** | 1.13 [1.01-1.27] | **0.035** |
| **Age >60 years** | 1.21 [1.10-1.34] | **<0.001** |
| **Non-white race** | 1.20 [1.09-1.32] | **<0.001** |
| **BMI, kg/m^2^ (increasing)** | 1.02 [1.01-1.03] | **<0.001** |
| **Diabetes** | 1.14 [1.04-1.27] | **0.010** |
| **Dialysis** | 2.02 [1.60-2.54] | **<0.001** |
| **Smoking history** | 1.17 [1.06-1.29] | **0.001** |
| **Prior cardiac surgery** | 1.34 [1.22-1.48] | **<0.001** |
| **Creatinine ≥2.0 mg/dL** | 1.62 [1.37-1.92] | **<0.001** |
| **Total bilirubin ≥3.0 mg/dL** | 2.14 [1.75-2.60] | **<0.001** |
| **PVR >3 WU** | 1.23 [1.11-1.36] | **<0.001** |
| **Days on waiting list (increasing)** | 1.00 [1.00-1.00] | 0.092 |
| **New allocation era (≥10/18/2018)** | 1.15 [1.04-1.26] | **0.006** |
| **Donor age ≥55 years** | 1.27 [1.06-1.53] | **0.009** |
| **Donor death due to stroke** | 1.18 [1.05-1.33] | **0.004** |
| **Ischemic time >6 hours** | 1.44 [1.10-1.90] | **0.009** |
| **Center ECD HT volume (cases/year, increasing)** | 1.00 [1.00-1.00] | 0.614 |

Multivariable hazard ratios with 95% confidence interval (CI) and p-value for interaction were calculated for association with 3-year graft failure. Bold type denotes p<0.05. BMI, body mass index; DSN, donor sequence number; ECD, extended criteria donor; HT, heart transplant; PVR, pulmonary vascular resistance; WU, Wood units. Log likelihood ratio for the multivariable Cox proportional-hazards model is -16084.98.
